# National Medical Convention in the City of New York

**Published:** 1845-11

**Authors:** 


### THE WESTERN JOURNAL.

NEW SERIES.—VOL. IV.—NO. V.

LOUISVILLE, NOVEMBER 1, 1845.

NATIONAL MEDICAL CONVENTION IN THE CITY OF NEW YORK.

We have received, and take great pleasure in laying before our readers, the following document:

"*National Convention of Physicians.*—The following preamble and resolutions, submitted by Dr. Davis, were adopted by the New York State Medical Society, at its late meeting:

"*Whereas*, It is believed that a National Convention would be conducive to the elevation of the standard of medical education in the United States, and

"*Whereas*, There is no mode of accomplishing so desirable an object, without concert of action on the part of the Medical Societies, Colleges, and Institutions of all the States. Therefore,

"*Resolved*, That the New York State Medical Society earnestly recommend a National Convention of delegates from medical societies and colleges in the whole Union—to convene in the city of New York, on the first Tuesday in May, in the year 1846, for the purpose of adopting some concerted action on the subject set forth in the foregoing preamble."

The project of a national convention, for the elevation of the medical profession in the United States, is not a novelty. Ten years ago, the medical convention of Ohio, declared the expediency of such an assemblage of delegates from all the schools of the Union, and sent its resolution to them, but the subject received no attention. Most ardently do we hope that the present proposal, emanating from the ancient and respectable medical society of the Empire State, will be responded to by all the schools and incorporated medical societies of the Union; and that in sending delegates, they will select wise and efficient men, who will have an eye single to the advancement of our beloved profession—rising above all merely local, temporary, and personal interests. We hope, also, that they will carry with them instructions on all the points which the respective corporations electing them may deem worthy of discussion and adoption.

The want of accordance among our schools, in any national system of instruction, it seems to us, could not well be greater than it is. Differences in the length of their sessions, in the number of their chairs, in the number of branches taught, in the price of their tickets, in their discipline, in the preparatory learning of their pupils, and in the attainments of candidates for graduation, without referring to other discrepancies, are matters worthy of the deepest consideration; to which we might add, the loose and very inadequate private pupilage through which the student passes, on his way to the school in which he proposes to attend lectures.

Still further, the question how far laws regulating the practice of physic should be encouraged, might be discussed; as well as their provisions, if they should be approved; so as to secure at least some approach to uniformity throughout the States.

Finally, the Convention might, perhaps, organize a National Medical Society for scientific improvement; which, meeting annually, in one of the great cities, might attract distinguished physicians from all parts of the Union; who should enlighten each other with the results of their experience, and the detail of their experimental researches; while, by intercommunion, they would animate each other to renewed exertion in the broad field of science, in which all may labor without exhausting the topics of inquiry in which it abounds.

In due time, we intend to renew this appeal to our brethren in the West, hoping, however, that enough has been already said to rouse them into preliminary action. D.

